# Barriers and Facilitators to Implementing Cost-Effective Evidence-Based Childhood Cancer Treatment in a Resource-Limited Setting in Egypt: A Qualitative Interview Study

**DOI:** 10.1200/GO.22.00424

**Published:** 2023-06-08

**Authors:** Ranin Soliman, Carl Heneghan, Anne-Marie Boylan, Jason Oke, Wael Eweida, Alaa Elhaddad

**Affiliations:** ^1^ Department of Continuing Education, University of Oxford, Oxford, United Kingdom; ^2^ Health Economics and Value Unit, Children's Cancer Hospital 57357 Egypt (CCHE), Cairo, Egypt; ^3^ Nuffield Department of Primary Care Health Sciences, Centre for Evidence-Based Medicine, University of Oxford, Oxford, United Kingdom; ^4^ Chief Operating Office, Children's Cancer Hospital 57357 Egypt (CCHE), Cairo, Egypt; ^5^ Paediatric Oncology Department, Children's Cancer Hospital 57357 Egypt (CCHE), Cairo, Egypt; ^6^ Paediatric Oncology Department, National Cancer Institute, Cairo University, Cairo, Egypt

## Abstract

**PURPOSE:**

Childhood cancer treatment is complex, resource-intensive, and expensive, and resource-limited settings would benefit from providing cost-effective treatment approaches on the basis of evidence. Effective implementation of cost-effective evidence-based treatment requires knowledge about factors influencing its use. In this study, we determined the clinicians' perceptions of the barriers and facilitators to implementing cost-effective evidence-based treatment for children with cancer in a resource-limited pediatric oncology setting in Egypt.

**METHODS:**

We conducted a qualitative study on the basis of semistructured interviews with senior clinicians who make high-level decisions on treatment protocols and tailored decisions for the atypically complicated group of patients. Purposive sampling was used to recruit the participants. Thematic analysis was conducted semantically to develop themes of barriers and facilitators.

**RESULTS:**

Fourteen participants agreed to participate in the study: nine pediatric oncologists; three surgeons; and two radiation oncologists. We identified four main themes of barriers and facilitators: awareness and orientation; knowledge, skills, and attitudes; system, resources, and context; and clinical practice. The main barriers included absence of easily available costs/cost-effectiveness data, limited resources and inability to pay for expensive novel (cost-effective) drugs, and gap between evidence and practice. The main facilitators included adopting standard treatment protocols on the basis of clinical effectiveness, leadership support, availability of patients' clinical and cost data from local context, and existing knowledge and skills in clinical research and health economic evaluation. The interview participants also provided suggestions to promote the implementation of cost-effective evidence-based treatment in priority areas.

**CONCLUSION:**

Our study findings provide an understanding of the barriers and facilitators affecting the implementation of cost-effective evidence-based treatment for childhood cancers in Egypt. We provide practical recommendations to address the implementation gaps with implications on practice, policy, and research.

## INTRODUCTION

Childhood cancer survival has improved to more than 80% in high-income countries, owing mainly to the significant progress in treatment over the years.^1^ This was achieved through cooperative multicenter clinical trials that have led to the development of evidence-based standard treatment protocols.^1^ Evidence-based practice (EBP) in pediatric oncology care is crucial to delivering optimal patient care.^2^ EBP involves adopting evidence-based resources, such as randomized trials, systematic reviews, or evidence-based guidelines, and using them in practice to make informed clinical decisions.^2^ A key step during applying EBP is to identify the implementation gaps between evidence and practice that may exist for many reasons, ranging from systems-related issues to individual factors such as limited awareness of the evidence.^3^

**CONTEXT**

**Key Objective**
This study aims to identify themes of barriers and facilitators to implementing cost-effective evidence-based childhood cancer treatment in Egypt. To our knowledge, this is the first study to explore the clinicians' perceptions in a resource-limited pediatric oncology setting in Egypt through in-depth interviews and thematic data analysis.
**Knowledge Generated**
We identified four themes of barriers and facilitators: awareness and orientation; knowledge, skills, and attitudes; system, resources, and context; and clinical practice. Main barriers included absence of easily available costs/cost-effectiveness data, limited resources, and gap between evidence and practice. However, the main facilitators included adopting standard treatment protocols, shared clinical decision making, and leadership support.
**Relevance**
Our findings provide helpful insights to tackle the existing barriers and address the implementation gaps to provide cost-effective evidence-based treatment in our clinical setting, and similar resource-limited settings in Egypt, and other low- and middle-income countries. This work provides participants' suggestions and recommendations from authors with clinical, policy, and research implications.

The treatment of children with cancer is complex, resource-intensive, and expensive, and resource-limited settings in low- and middle-income countries (LMICs) could benefit from providing cost-effective treatments that offer the greatest value for money (ie, improved health outcomes relative to costs). That is why, it is important to identify and implement the most cost-effective treatment strategies on the basis of the best available evidence in these resource-limited contexts. Effective implementation of evidence-based cost-effective childhood cancer treatment requires knowledge about the barriers and facilitators influencing its use.^4^ Therefore, it is crucial to understand the clinicians' perspectives of the factors that affect making informed clinical decisions and adopting cost-effective treatment approaches in an LMIC setting. This study aimed to determine the clinicians' perceptions of barriers and facilitators to implementing cost-effective evidence-based treatment for children with cancer in a resource-limited pediatric oncology setting in Egypt.

## METHODS

The study was designed and reported according to the Consolidated Criteria for Reporting Qualitative Research checklist,^5^ included in Appendix Table A1.

### Design, Setting, and Scope

We conducted a descriptive qualitative study on the basis of semistructured interviews guided by some predefined topics and open-ended questions. The study took place at the Children's Cancer Hospital Egypt (CCHE), a nonprofit pediatric oncology center in Egypt that provides comprehensive childhood cancer treatment free of charge to about 40%-50% of children with cancer in Egypt on the basis of donations.^6-9^ At CCHE, childhood cancer care is delivered through shared decision making in multidisciplinary teams for each childhood cancer type known as study teams, led by a senior pediatric oncologist/hematologist (study team leader), and includes surgeons, radiation oncologists, pathologists, radiologists, pharmacist(s), and clinical researcher(s). The study team is responsible for making high-level decisions such as the selection or modification of the standard treatment protocols.

Patients are treated according to standard protocols adopted from the Children's Oncology Group^10^ for the majority of cancers, and the St Jude Total XV protocol for acute lymphoblastic leukemia.^11^ The treatment protocols were chosen by the study teams on the basis of evidence of clinical effectiveness and feasibility of their adoption at CCHE, depending on resource availability. Some atypically complicated patients (whose management is not indicated in the treatment protocols) are discussed by senior clinicians in weekly multidisciplinary combined clinics, where tailored decisions are made about disease relapse/progression, treatment failure, or palliative care for noncurative intent. The main scope of this study was to focus on these two decision-making levels: (1) high-level decisions to select/modify treatment protocols, and (2) tailored decisions to manage atypically complicated cases. However, day-to-day individual decisions were outside the scope of this study.

### Participants and Recruitment

Purposive sampling was used to recruit senior clinicians who provide treatment for children with cancer at CCHE.^12^ The inclusion criteria included (1) senior clinicians involved in the high-stake/tailored decisions regarding the treatment of children with cancer (senior pediatric oncologists, radiation oncologists, and surgeons); (2) willingness and availability to participate in the study; and (3) providing written informed consent. However, exclusion criteria included less experienced young clinicians and other study team members who are not the main key players in the high-level decision-making process in our context. We aimed to conduct 12-15 interviews, with the final number of participants determined by data saturation.^12^ Recruitment of participants was done through an internal announcement at the hospital through e-mails and direct communication between the lead author (R.S.) and the potentially eligible participants.

### Data Collection

A narrative semistructured interview guide, which was developed by the lead author (R.S.) in consultation with another author (A.-M.B.), was used to determine the participants' perceptions (Appendix Table A2). Participants were asked about their perceptions of barriers and facilitators on the two decision-making levels, where the interviewed senior clinicians were part of both committees: study teams and combined clinics. Qualitative data were collected during the in-depth face-to-face interviews that took place between July 1 and September 5, 2022, at CCHE on a one-to-one basis. All interviews were conducted by R.S. (trained qualitative researcher), audio-recorded, translated from Arabic to English, and transcribed verbatim.

### Ethical Considerations

Ethics approval for the study was granted by the institutional review board at CCHE on September 9, 2021 (ref. number: CCHE-35) and by the Oxford Tropical Research Ethics Committee (OxTREC) for minimal risk studies at the University of Oxford on January 7, 2022 (OxTREC ref. 569-21). All participants provided written informed consent using the forms approved by the ethics committees of both institutions, to ensure all ethical considerations in qualitative research studies were met.^13^

### Thematic Data Analysis

Thematic analysis was conducted, where qualitative data were analyzed semantically and inductive analysis was performed with full presentation of themes.^14^ Data coding was done without trying to fit into a pre-existing coding frame or the researchers' analytic preconceptions, and thematic analysis was data-driven.^14^ We conducted data analysis using NVivo (version 1.6.1) software to facilitate coding.^15^ Data analysis was conducted concurrently with data collection, refined throughout the interview process, then validated and finalized upon completion of all interviews in an iterative process. The lead author (R.S.) categorized codes of similar concepts/perceptions into related domains and discussed them with the other two authors (A.-M.B. and C.H.) to develop the final themes. Supporting quotes were chosen to demonstrate the developed themes. We performed subgroup analyses by level of decision making (high-level *v* tailored decisions), and health profession of included clinicians (pediatric oncologists, surgeons, and radiation oncologists).

## RESULTS

### Characteristics of Participants

Fourteen participants were interviewed of the 15 invited senior clinicians; one refused to participate because of unavailability. Interviews lasted between 30-75 minutes, and none were repeated. Data saturation was reached after conducting 12 interviews and was confirmed by adding two more interviews, after which further data collection would be redundant. Table 1 shows the characteristics of the interviewed senior clinicians, namely nine pediatric oncologists/hematologists (study team leaders), three surgeons, and two radiation oncologists. Most participants (9/14; 64.3%) were male, and the mean age of all participants was 45 years (range, 42-55 years). Years of clinical experience ranged between 15 and 25 years in the different specialties. To maintain data confidentiality, further participants' details were not included as they all work in the same hospital setting and could be easily identifiable.

**TABLE 1:**
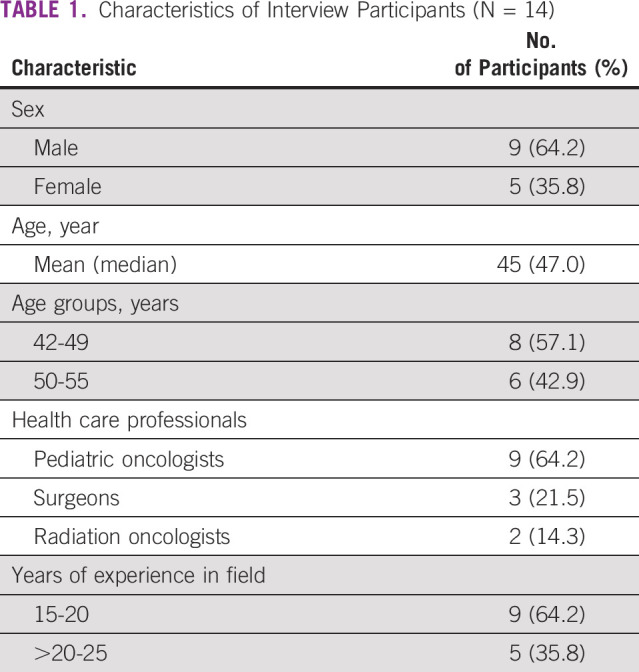
Characteristics of Interview Participants (N = 14)

### Themes of Barriers and Facilitators

**FIG 1:**
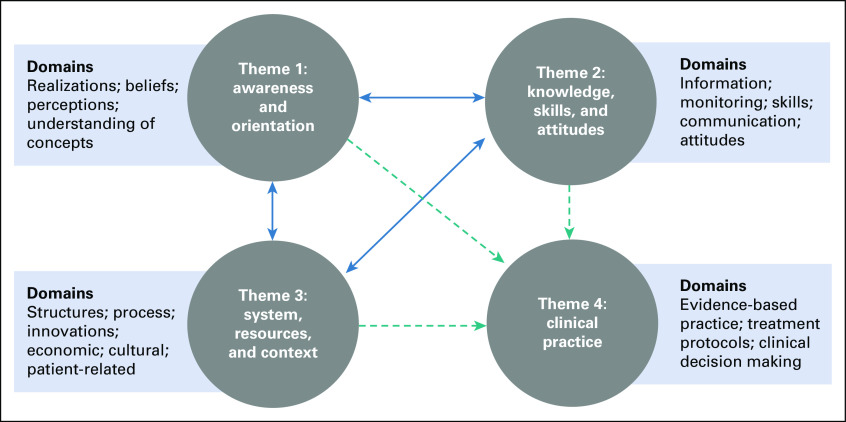
Relationships between core themes of barriers and facilitators to implementing cost-effective evidence-based treatment for children with cancer. The arrows show the inter-relationships among the different themes of barriers and facilitators. Solid blue arrows are bidirectional, showing mutual relationships among themes 1, 2, and 3 affecting one another. Dotted teal arrows are unidirectional, showing one-way relationships where themes 1, 2, and 3 affect theme 4.

The following four themes captured the clinicians' perceptions of barriers and facilitators to implementing cost-effective evidence-based treatment for children with cancer at CCHE/Egypt: (1) awareness and orientation; (2) knowledge, skills, and attitudes; (3) system, resources, and context; and (4) current practice. Table 2 presents an overview of the themes and domains of barriers and facilitators reported by all study participants across the two decision-making levels of interest. Figure 1 illustrates the inter-relationships among the four themes. Most of the barriers and facilitators were stated by pediatric oncologists addressing the four developed themes, as they represent the majority of participants. Subgroup analysis by health profession showed that surgeons and radiation oncologists addressed barriers under only two themes: knowledge, skills, and attitudes; and systems, resources, and context (Appendix Table A3). However, subgroup analysis by decision-making level identified distinct barriers and facilitators under each theme (Appendix Table A4). There were no differences in participants' perceptions on the basis of sex, age, or years of experience.

**TABLE 2:**
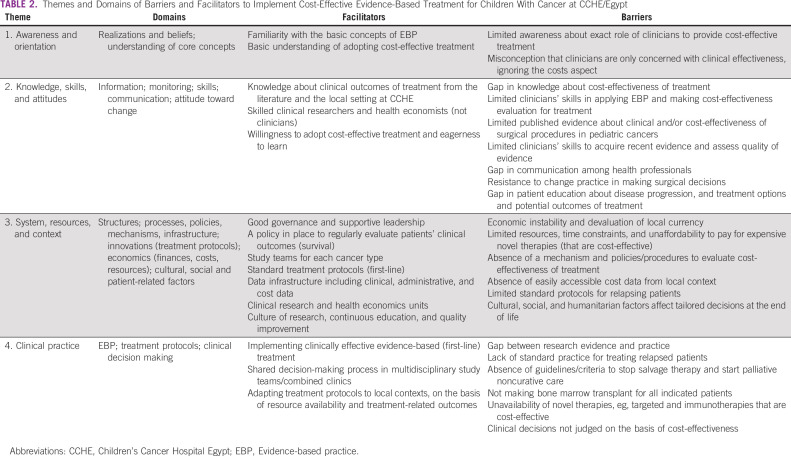
Themes and Domains of Barriers and Facilitators to Implement Cost-Effective Evidence-Based Treatment for Children With Cancer at CCHE/Egypt

#### Theme 1: Awareness and orientation.

Familiarity with basic EBP concepts and awareness about the importance of implementing cost-effective treatment were important facilitators highlighted by the clinicians. Most participants reported that they follow standard treatment protocols on the basis of evidence, quoting “We treat our children with cancer based on standard protocols adapted from international protocols based on best available evidence of clinical effectiveness” (PO–01). However, their lack of awareness about their active roles in selecting cost-effective treatment strategies was a common barrier among them. Many of them had a misconception that clinicians should only focus on the clinical benefit of treatment, whereas the costs and financial aspects are not their responsibility, but are the decisions of the hospital management. This was quoted as “Clinicians should only be concerned with the clinical outcomes of treatment, while decisions about the costs of care should be taken by the hospital's upper management” (PO–04). Only a small number of participants reported their understanding of their essential roles in judging the cost-effectiveness of treatment interventions in the clinical decision-making process, quoting “We should also make tailored decisions in the combined clinics based on cost-effectiveness of treatment” (PO–9).

#### Theme 2: Knowledge, skills, and attitudes.

Some participants indicated that adequate knowledge of the clinical outcomes of treatment on the basis of evidence from the literature and the local setting were key facilitators. This was quoted as “We have an advantage at CCHE because we can generate local knowledge about patients' outcomes from real-world data” (PO–5). However, the absence of easily accessible cost data and the lack of knowledge about cost-effectiveness of treatment from local context were commonly reported barriers, quoting “Our biggest problem is that clinicians do not have access to data about how much the treatment costs at CCHE, so we can't decide whether treatment would be cost-effective or not” (PO–3). One surgeon reported that there is limited published literature about the cost-effectiveness of surgical interventions, quoting “There is limited published literature about clinical and/or cost-effectiveness of surgical procedures in pediatric cancers that is relevant to the patients we see in practice” (PS–01). Although many clinicians mentioned that they lack the necessary skills to understand cost-effectiveness analyses, they were eager to learn how to interpret the findings and willing to change practice, accordingly, quoting “We don't know how to make cost-effectiveness analyses or understand their results” (RO–1).

#### Theme 3: System, resources, and context.

The main facilitators under this theme included supportive leadership, existing study team groups, standard treatment protocols, clinical/cost data infrastructure, a policy in-place to regularly evaluate patients' clinical outcomes, and an established research department and health economics unit. One clinician commented “We have the necessary structures and tools to provide evidence-based treatment, and we should use the existing resources to make it cost-effective as well” (PO–06). On the other hand, most participants mentioned that a main barrier was the absence of a mechanism/system to regularly monitor cost-effectiveness of treatment at CCHE, quoting “We don't have any written policies to evaluate cost-effectiveness of treatment, and no mechanisms exist about how, when, or who should be involved in the process” (PO–04).

Other significant barriers included limited resources, time constraints, and inability to pay for the expensive (but cost-effective) novel therapies. One of the surgeons commented “We do not have adequate time or resources to evaluate and compare the cost-effectiveness of different surgical interventions” (PS–2). Furthermore, patient-related sociocultural and humanitarian factors were perceived as barriers to providing cost-effective care while making tailored decisions for relapsing/progressive disease, quoting “Sometimes patients put pressure on doctors to provide additional salvage therapy for terminally ill relapsed patients, believing that it will save their lives, when they should only receive palliative end-of-life care” (PO–2).

#### Theme 4: Clinical practice.

Participants reported that adopting evidence-based standard protocols for first-line therapy and shared decision making in multidisciplinary teams were key facilitators. However, existing implementation gap between evidence from real-world data and current practice was considered a major problem, as quoted “We conduct research studies using real-world data at CCHE, but we do not use research findings to improve clinical practice” (PO–8). Additionally, current practice for managing relapsing/progressive patients does not follow (cost-effective) evidence-based protocols for the majority of cancers, and there does not exist well-documented guidelines regarding criteria for discontinuing salvage therapy for nonresponding patients and initiating palliative care for noncurative intent. This was supported by the quote, “In case of relapsing AML patients who could not undergo bone marrow transplant, recent evidence suggests that it is not cost-effective to provide many cycles of intensive salvage chemotherapy. Yet, this happens while making tailored decisions” (PO–2).

#### Participants' suggestions.

The participants provided suggestions to address the existing barriers (presented in Table 3) classified under the same developed themes. A key recommendation was to achieve good governance by developing mechanisms and processes to ensure the evaluation and implementation of evidence-based cost-effective treatment, quoting “Cost-effectiveness of treatment should be regularly evaluated in study teams, and a committee should be formed to oversee the implementation” (PO–7). Others suggested making cost/cost-effectiveness data available to clinicians, quoting “We need easy access to cost/cost-effectiveness data for the different treatment interventions to help us make informed clinical decisions” (RO–2).

**TABLE 3:**
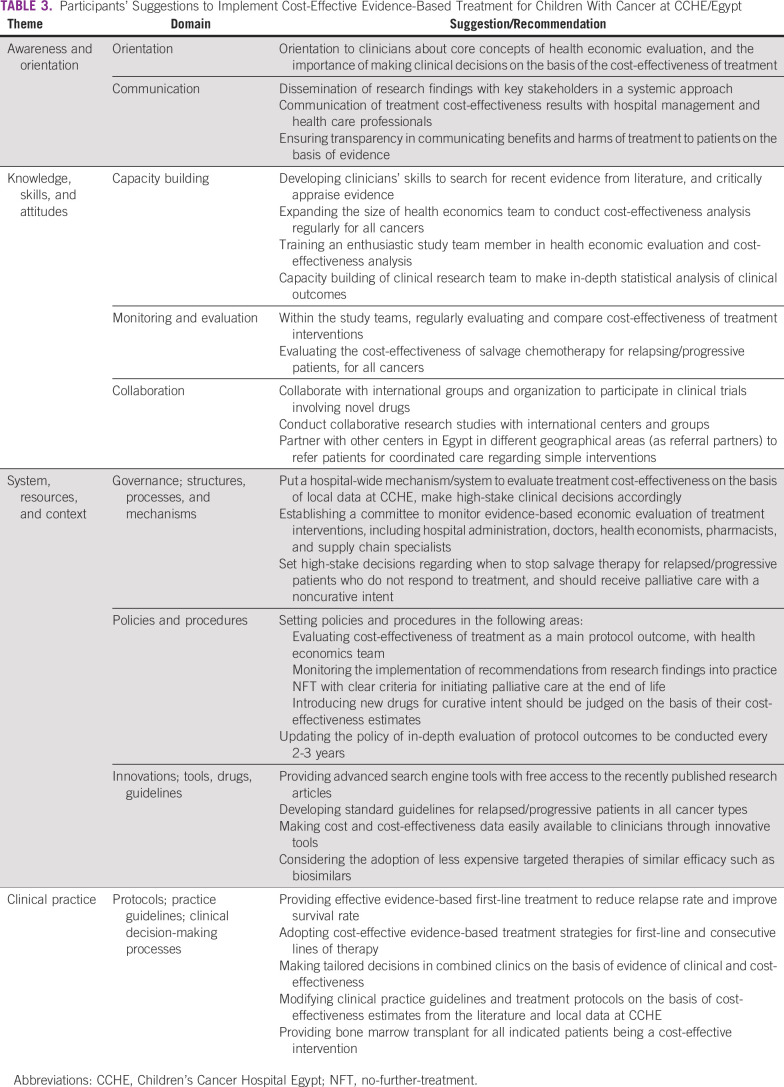
Participants' Suggestions to Implement Cost-Effective Evidence-Based Treatment for Children With Cancer at CCHE/Egypt

## DISCUSSION

In this study, we identified four themes of barriers and facilitators to implementing cost-effective evidence-based childhood cancer treatment in Egypt/CCHE. To our knowledge, this is the first study to determine the clinicians' perceptions of barriers and facilitators in a resource-limited pediatric oncology setting in Egypt through in-depth interviews and thematic data analysis.

The primary facilitators were the adoption of standard treatment protocols shared decision making, availability of clinical and cost data infrastructures, supportive leadership, presence of trained health economists, and favorable clinician attitudes toward change. However, gap in knowledge about the cost/cost-effectiveness of treatment, limited resources, inability to pay for novel expensive (cost-effective) therapies, and absence of standard practice guidelines for relapsed patients were significant barriers. The participants suggested key effective solutions to address the existing barriers, such as developing a hospital-wide system/mechanism with written policies for evaluating and implementing cost-effective evidence-based treatment.

Pediatric oncologists reflected on barriers and facilitators under the four developed themes, while surgeons and radiation oncologists only addressed two themes of barriers (Appendix Table A3). Gender balance was not disregarded in selection of participants; it is representative of the pool of senior clinicians in our setting, which has fewer females in senior decision-making positions, with all surgeons being male.

The developed themes of barriers and facilitators in our study were similar to those identified in the literature; however, no published studies were relevant to implementing cost-effective evidence-based treatment for childhood cancers. Several articles reported on the barriers and facilitators to implementing EBP; however, none of these focused on childhood cancer care.^16-19^ A systematic review by Bach-Mortensen et al^20^ found that limited resource availability was the main barrier to implementing evidence-based interventions, while organizational culture and proper implementation strategies were common facilitators.

According to Sugalski et al,^21^ the primary barriers to implementing evidence-based supportive care guidelines in pediatric oncology were gaps in knowledge and administrative hurdles, while the main facilitators were willingness to change and implementation mechanisms. As reported in our results (Appendix Table A3), barriers to EBP implementation by surgeons included a lack of EBP skills, time constraints, and limited published evidence in pediatric oncology surgery.^22^ The main barriers to evaluate the health economic benefit (cost-effectiveness) of interventions in the Middle Eastern countries were the limited access to clinical effectiveness and cost data from local contexts, lack of expertise and awareness, and absence of national governing bodies.^23,24^

Despite the strengths of this study, some limitations exist. First, this study was conducted in a single pediatric oncology setting in Egypt where transferability to other settings could be limited by the predominance of the hospital's context and culture. Nevertheless, some of these factors could apply to similar resource pediatric oncology settings in Egypt or other LIMICs. Second, since the majority of participants were pediatric oncologists, the study sample could be weighted toward their views. However, childhood cancer care is predominantly composed of pediatric oncologists, with fewer surgeons and radiation oncologists involved. Third, there remains a potential risk for participants to be identifiable as they work in the same hospital. However, participation was voluntary and they signed informed consent ensuring data confidentiality. Fourth, although direct translation from Arabic to the English language was conducted during transcription, other semantic meanings may not have been captured.

Generating qualitative evidence from experts' opinions on the basis of local clinical experience will help translate knowledge into practice and address the implementation gaps. The findings from this study would have practice and policy implications on different levels: committee level; hospital level; national level; and regional/context-relevant level. On the committee level (tailored decisions for atypical/complicated patients) and the hospital level (high-level decisions about treatment protocols), capitalizing on existing facilitators and tackling current barriers, guided by the participants' suggestions, would help develop organization-wide mechanisms and set effective policies to implement cost-effective evidence-based treatment in practice. Accordingly, this would enable clinicians to make better informed clinical decisions considering cost-effective approaches. On the national and regional/context-relevant levels, the findings from our study are context-specific, where our unique findings and practical recommendations could be extrapolated to other resource-limited pediatric oncology settings in Egypt or other LMICs.

We provide the following specific recommendations to address the implementation gaps.

1. Develop an online platform including recent evidence about cost-effective childhood cancer treatment.
2. Apply the Ottawa Model of Research Use knowledge translation framework to transfer research-based knowledge into practice.
3. Prioritize key participants' suggestions and develop an action plan for implementation.
4. Understand the perceptions of young clinicians about barriers/facilitators to implement cost-effective evidence-based treatment in day-to-day decisions.
5. Determine expert opinions of other key stakeholders (patients/families, hospital managers, and payers) to implement cost-effective evidence-based treatment.
6. Study the perceptions of patients' families toward communication about end-of-life care.

In conclusion, our findings provide an important understanding and adds to the knowledge base about barriers and facilitators related to implementing cost-effective evidence-based treatment strategies in a resource-limited pediatric oncology setting in Egypt. We provide participants' suggestions and specific recommendations from authors to implement cost-effective evidence-based treatment with clinical, policy, and research implications.

## Figures and Tables

**TABLE A1:**
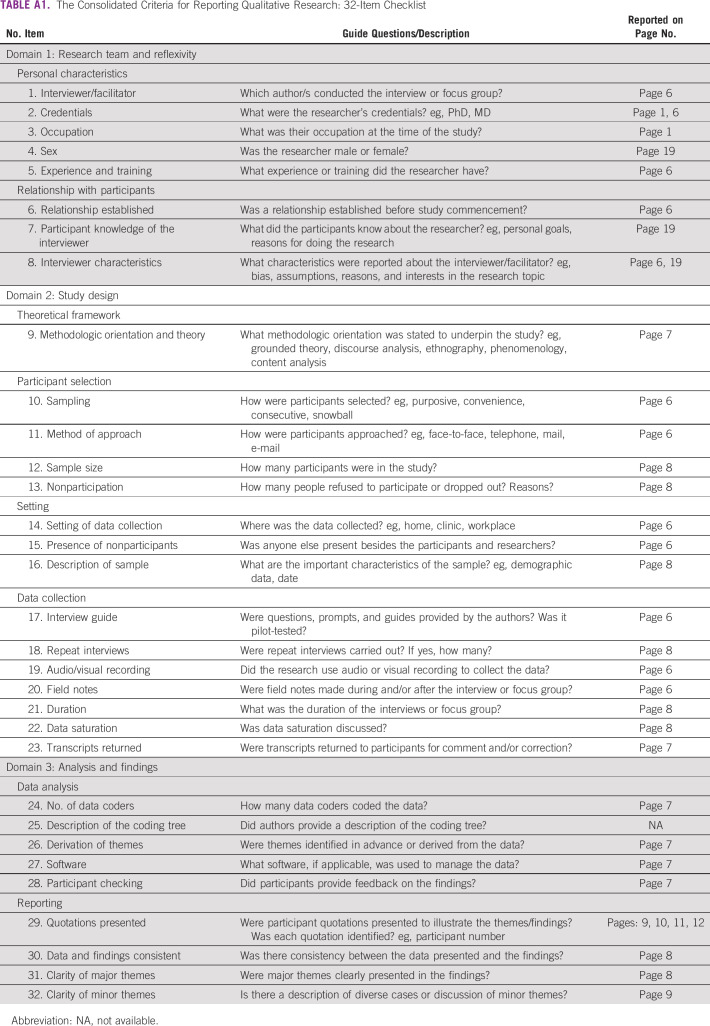
The Consolidated Criteria for Reporting Qualitative Research: 32-Item Checklist

**TABLE A2:**
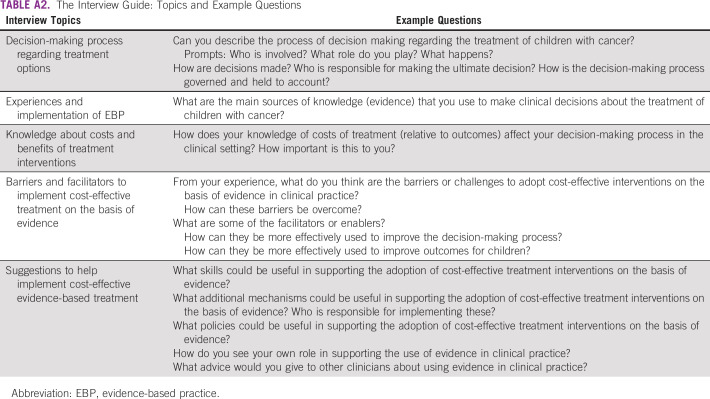
The Interview Guide: Topics and Example Questions

**TABLE A3:**
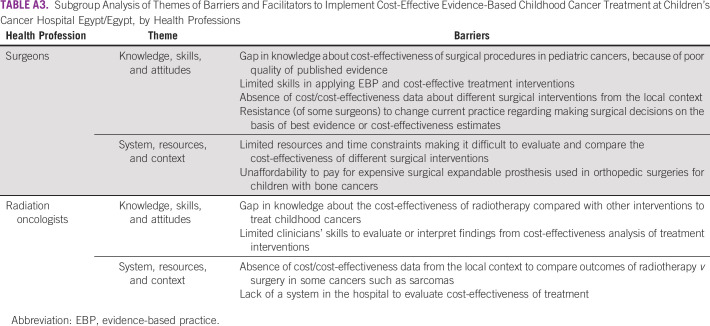
Subgroup Analysis of Themes of Barriers and Facilitators to Implement Cost-Effective Evidence-Based Childhood Cancer Treatment at Children's Cancer Hospital Egypt/Egypt, by Health Professions

**TABLE A4:**
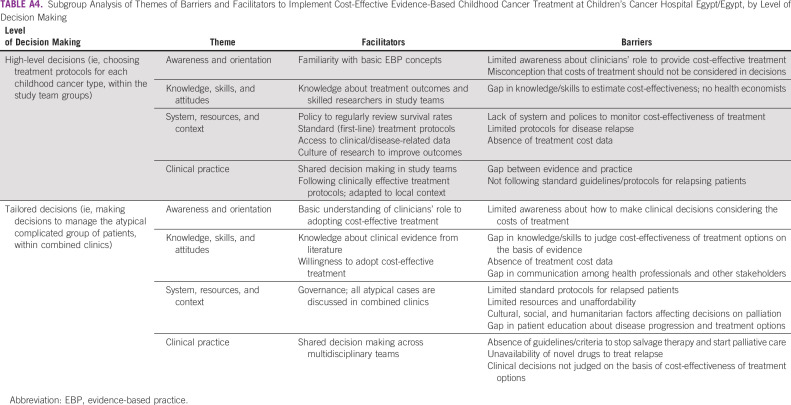
Subgroup Analysis of Themes of Barriers and Facilitators to Implement Cost-Effective Evidence-Based Childhood Cancer Treatment at Children's Cancer Hospital Egypt/Egypt, by Level of Decision Making
